# Graphene Oxide: Key to Efficient Charge Extraction and Suppression of Polaronic Transport in Hybrids with Poly (3-hexylthiophene) Nanoparticles

**DOI:** 10.1021/acs.chemmater.3c00008

**Published:** 2023-04-20

**Authors:** Eduardo Colom, Javier Hernández-Ferrer, Alejandro Galán-González, Alejandro Ansón-Casaos, Mario Navarro-Rodríguez, Elisa Palacios-Lidón, Jaime Colchero, Javier Padilla, Antonio Urbina, Raul Arenal, Ana M. Benito, Wolfgang K. Maser

**Affiliations:** † Instituto de Carboquímica (ICB-CSIC), E-50011 Zaragoza, Spain; ‡ Centro de Investigaciones Científicas Avanzadas, Universidade da Coruña (CICA), E-15008 San Vicenzo de Elviña, A Coruña, Spain; § Departamento de Física, Edificio CIOyN, Universidad de Murcia, E-30100 Murcia, Spain; ∥ Departamento de Física Aplicada y Tec. Naval, Universidad Politécnica de Cartagena, E-30202 Cartagena, Spain; ⊥ Departamento de Ciencias e Instituto de Materiales Avanzados y Matemáticas (INAMAT^2^), Universidad Pública de Navarra (UPNA), E-31006 Pamplona, Spain; # Instituto de Nanociencia y Materiales de Aragón (INMA-CSIC-Univ. Zaragoza), E-50009 Zaragoza, Spain; ∇ ARAID Foundation, E-50018 Zaragoza, Spain; ▼ Laboratorio de Microscopias Avanzadas (LMA)Universidad de Zaragoza, E-50018 Zaragoza, Spain

## Abstract

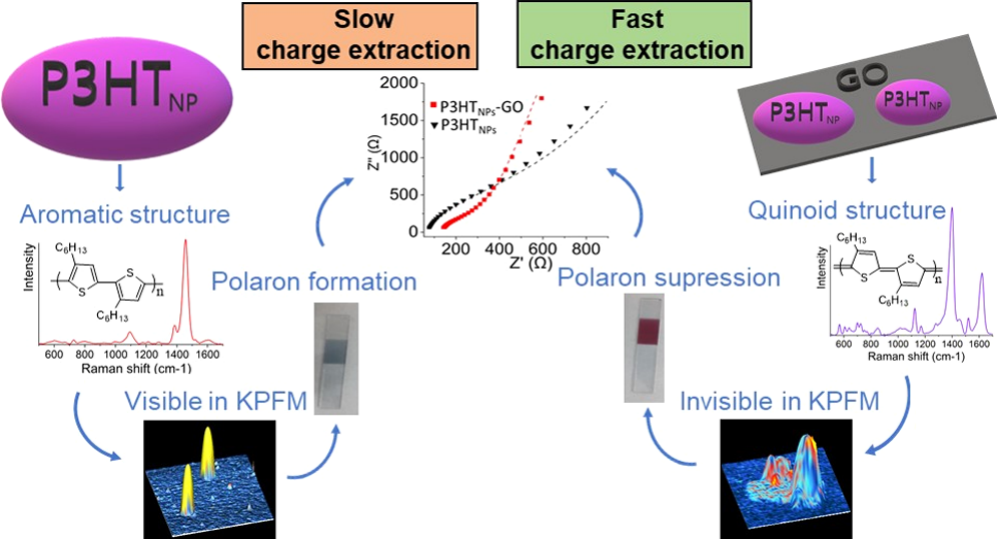

Nanoparticles (NPs) of conjugated polymers in intimate contact with sheets of graphene oxide (GO) constitute a promising class of water-dispersible nanohybrid materials of increased interest for the design of sustainable and improved optoelectronic thin-film devices, revealing properties exclusively pre-established upon their liquid-phase synthesis. In this context, we report for the first time the preparation of a P3HT_NPs_–GO nanohybrid employing a miniemulsion synthesis approach, whereby GO sheets dispersed in the aqueous phase serve as a surfactant. We show that this process uniquely favors a quinoid-like conformation of the P3HT chains of the resulting NPs well located onto individual GO sheets. The accompanied change in the electronic behavior of these P3HT_NPs_, consistently confirmed by the photoluminescence and Raman response of the hybrid in the liquid and solid states, respectively, as well as by the properties of the surface potential of isolated individual P3HT_NPs_–GO nano-objects, facilitates unprecedented charge transfer interactions between the two constituents. While the electrochemical performance of nanohybrid films is featured by fast charge transfer processes, compared to those taking place in pure P3HT_NPs_ films, the loss of electrochromic effects in P3HT_NPs_–GO films additionally indicates the unusual suppression of polaronic charge transport processes typically encountered in P3HT. Thus, the established interface interactions in the P3HT_NPs_–GO hybrid enable a direct and highly efficient charge extraction channel via GO sheets. These findings are of relevance for the sustainable design of novel high-performance optoelectronic device structures based on water-dispersible conjugated polymer nanoparticles.

## Introduction

Semiconducting π-conjugated polymers critically contribute to the development of the field of organic electronics.^1^ Their tunable optical and electronic properties combined with low-cost liquid-phase processing possibilities enable the fabrication of layered optoelectronic thin-film platforms constituting the core in organic field-effect transistors (OFETs),^2^ organic photovoltaics (OPV),^3^ light-emitting devices (OLEDs),^4^ and electrochromic applications.^5^ A benchmark conjugated polymer, being used as a photoactive layer in these applications, is poly(3-hexylthiophene) (P3HT).^6^ Processed into films, its polymer chains are able to crystallize, forming aggregate structures. These nanocrystalline aggregates have a significant influence on the optoelectronic, charge transfer, and charge transport properties of the resulting P3HT films.^7,8^ Controlling film fabrication parameters,^9^ applying thermal post-treatment steps,^10^ making use of donor–acceptor blends,^11^ and exploiting chemical doping^12^ or electrochemical oxidation approaches^13^ are widely studied strategies to influence the nanocrystalline aggregate structure and their effects on the exciton^14^ and polaron dynamics^15^ and to establish favorable interface interactions^16^ to achieve enhanced device performance of layered film devices. More recently, self-assembly strategies shaping conjugated polymers into nanofibers or spherical nanoparticles have enjoyed increased interest.^17−21^ They convey the advantage that the aggregation structure of the polymer chains in these nanoparticles is exclusively pre-established upon their formation in the liquid phase and that the resulting optoelectronic properties are not being altered when processed into films, thus eliminating tedious tasks of controlling film formation parameters.^22^ Moreover, the liquid phase approaches, involving either emulsion or reprecipitation technologies, provide a unique opportunity to create stable aqueous dispersions of conjugated polymer nanoparticles and environmentally friendly processing possibilities.^19^ In addition, the self-assembly routes facilitate exciting pathways to interface P3HT_NPs_ with different kinds of functional materials and to design novel nanohybrid materials, such as donor–acceptor core–shell nanoparticles of P3HT–PCBM^23^ and P3HT–CdSe^24^ or charge transfer complexes based on P3HT_NPs_ and transition-metal dichalcogenides, such as P3HT_NPs_–MoS_2_ and P3HT_NPs_–WS_2_.^25^ A functional platform of special interest for optoelectronic applications is the use of graphene oxide (GO).^26,27^ Providing tunable conductive properties, it enables improved charge transfer properties and offers promise as a transport layer for electrons and holes in OPV devices.^28^ Furthermore, being of amphiphilic character, GO suitably can be used in liquid-phase self-assembly approaches of conjugated polymers to establish improved interface interactions.^28,29^ More recently, we have shown that the presence of GO sheets, used as an additive in the water phase of a reprecipitation synthesis process, uniquely favors the formation of P3HT chains with an enhanced planar conformation upon the liquid-phase self-assembly process.^30,31^ This game-changing role of GO thus facilitates the enhanced development of J-aggregates inside the P3HT_NPs_, being responsible for establishing close interface interactions between P3HT_NPs_ located on GO sheets and the creation of a water-soluble P3HT_NPs_–GO charge transfer complex with enhanced optoelectronic properties. While in situ reprecipitation processes thus successfully exploit the presence of GO sheets, the influence of GO in emulsion-based self-assembly technologies, which intrinsically require the additional presence of a stabilizing surfactant, on the growing P3HT nanoparticles and their properties needs to be explored.

In this work, we use for the first time a miniemulsion process whereby GO, due to its amphiphilic character, serves as a surfactant additive in the aqueous phase. The morphology and electronic properties of the resulting sample are probed in the liquid phase, the solid state in the form of individual objects, and as films. Transmission electron microscopy (TEM) and atomic force microscopy (AFM) studies confirm the formation of a P3HT_NPs_–GO nanohybrid consisting of P3HT_NPs_ in intimate contact with GO sheets. UV–vis and fluorescence spectroscopy techniques suggest important charge interface interactions between P3HT_NPs_ and GO established upon the miniemulsion process, while Raman spectroscopy demonstrates significant changes in the P3HT chain conformation of P3HT_NPs_ toward a quinoid-like structure in the hybrid material. Studies of the surface potential carried out by Kelvin probe force microscopy (KPFM) on isolated individual P3HT_NPs_–GO nanostructures show a distinct electronic response of the surface potential of P3HT_NPs_ attached to GO sheets in the hybrid compared to that of P3HT_NPs_. Cyclic voltammetry and electrochemical impedance spectroscopy analyses of the nanohybrid films reveal an efficient and fast charge extraction process when compared to that of pure P3HT_NPs_. The electrochromic behavior of P3HT_NPs_–GO films suggests that the established interface interaction between P3HT_NPs_ and GO serves as a preferred and efficient charge extraction channel, thereby suppressing the polaronic charge transport process typically encountered in P3HT_NPs_. This study thus underlines the importance of the applied synthesis approach for controlling interface interaction and establishing favorable charge transport/extraction properties in hybrids of conjugated polymer nanoparticles and sheets of graphene oxide.

## Results and Discussion

**Figure 1:**
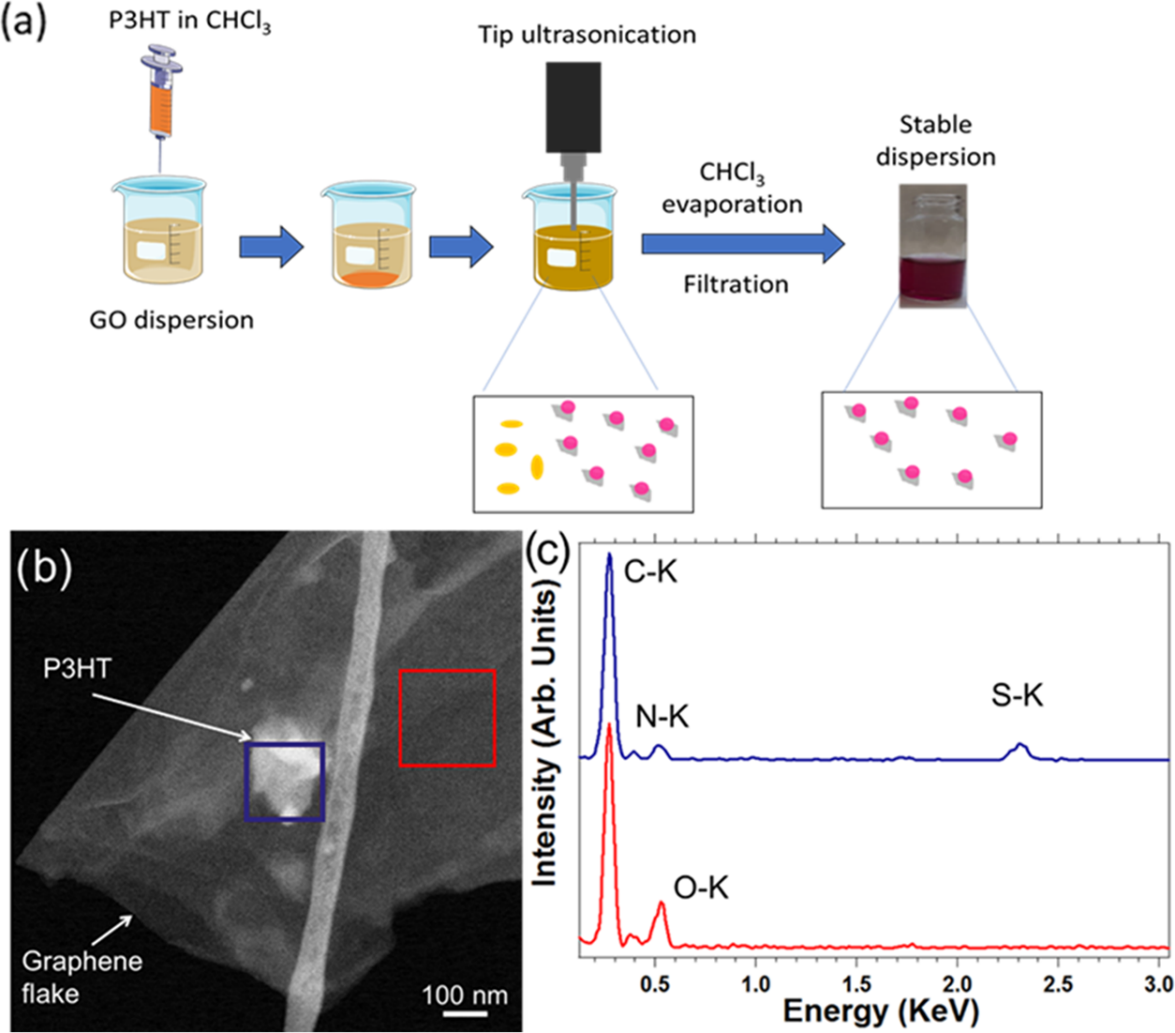
(a) Scheme of the employed P3HT_NPs_–GO miniemulsion synthesis route. (b) HAADF-STEM image of a P3HT_NP_–GO hybrid nanostructure. (c) EDS spectra of a region related to the P3HT_NP_ located on GO (blue area in (b)) and of a zone related to GO alone (red area in (b)).

The P3HT_NPs_–GO nanohybrid material is prepared by applying a miniemulsion synthesis approach using, for the first time, GO sheets as a surfactant in the aqueous phase. This results in stable water dispersions of the resulting P3HT_NP_–GO hybrid. Figure 1a shows the scheme of the preparation approach, while the synthesis details and the critical dependence of hybrid formation on the GO sheet size are described in the Experimental Section and Supporting Information, respectively.

Transmission electron microscopy (TEM) investigations provide local information on the hybrid nanostructures, see Figure 1b,c. A GO flake of around 1 μm in size is depicted in the high-angle annular dark-field (HAADF) STEM image in Figure 1b, and a structure (brighter area) is visible in the middle of this flake. Since the intensity of these HAADF images is proportional to the density of the material and the atomic number of the elements present in the analyzed region,^32,33^ it is reasonable to assume that the structure located on the GO flakes corresponds to a P3HT_NP_ nanoparticle. Indeed, energy dispersive X-ray (EDS) STEM analyses performed in parallel (Figure 1c) clearly reveal the presence of sulfur in this structure (see the blue highlighted square area in Figure 1b), which is absent in the region of the pristine GO flake (see the red highlighted square area in Figure 1b). These observations thus confirm that the P3HT_NP_–GO hybrid is composed of P3HT_NPs_ located on GO sheets.

**Figure 2:**
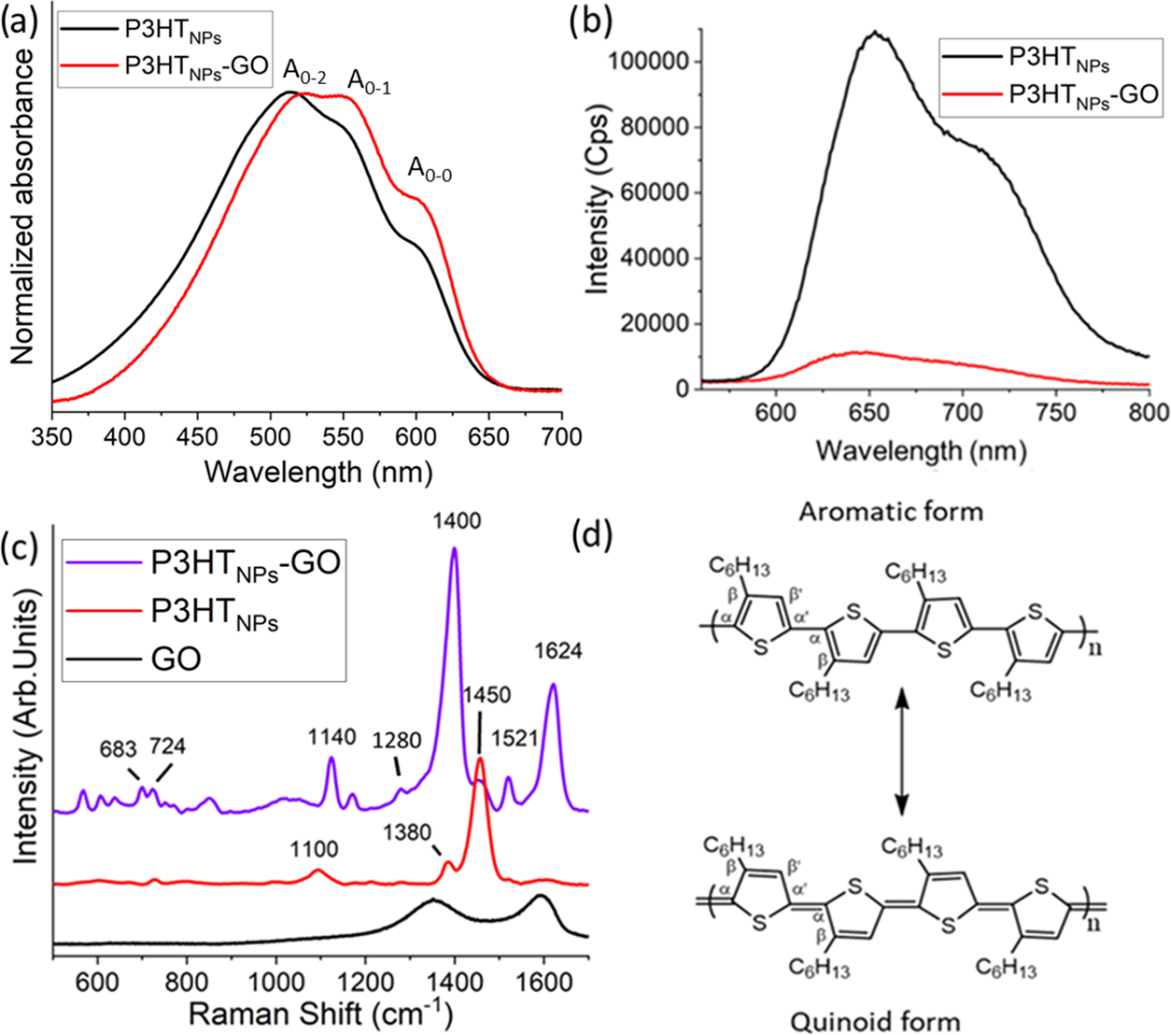
(a) Normalized background-corrected UV–vis absorption spectra of P3HT_NPs_ and P3HT_NPs_–GO. (b) Photoluminescence emission spectra of P3HT_NPs_ and P3HT_NPs_–GO. (c) Raman spectra of GO, P3HT_NPs_, and P3HT_NPs_–GO. (d) Aromatic and quinoid forms of P3HT.

The liquid-phase properties provide the first insight into the electronic properties and are probed by UV–vis and photoluminescence spectroscopy. UV–vis spectra of P3HT_NPs_ and P3HT_NPs_–GO (Figure 2a) show a broad π–π* transition absorption band ranging from 350 to 700 nm, exhibiting a series of three well-resolved peaks at 520, 560, and 620 nm, corresponding to the fundamental vibronic A_0-2_, A_0-1_, and A_0-0_ transitions, indicative of the presence of excitonic coupling between the P3HT chains.^34−37^ The intensity ratio between the A_0-0_ and A_0-1_ transitions, as determined from the normalized background-corrected spectra (see the Supporting Information for original data and background correction), provides important information on the internal arrangement of the polymer chains. A value of 0.55 for the case of P3HT_NPs_ reveals a polymer chain structure typical for the formation of H-aggregates, whereby the P3HT chains acquire a face-to-face orientation, exhibiting interchain interactions. This ratio increases to 0.65 for the case of P3HT_NPs_–GO. While this value still is indicative of the presence of H-aggregates, its enhancement, associated with a decrease in the exciton coupling constant from 40 to 30 meV (see the Supporting Information, Section S3), implies either a decrease in the interchain stacking order and/or an increase in the intrachain interactions,^34,35^ being facilitated by a higher degree of planarity of the polymer chain backbone.^38^ This is most likely induced by the presence of GO and provides a first hint for establishing intimate interface interactions between P3HT_NPs_ and GO in the nanohybrid influencing on the electronic structure of P3HT_NPs_.

In Figure 2b, the photoluminescence (PL) emission spectrum of P3HT_NPs_ is characterized by two intense peaks at 650 and 710 nm. Its intensity decreases by 90% in the P3HT_NPs_–GO nanohybrid. Such a significant quenching effect generally is associated with the existence of charge transfer processes between the P3HT polymer chains of the carbon nanostructure.^39^ Therefore, UV–vis and PL spectroscopy techniques clearly point to the formation of a strong interaction between the polymer chains of P3HT_NPs_ and GO.

To study these interactions in the hybrid material in more detail, Raman spectroscopy analyses were performed on GO, P3HT_NPs_, and P3HT_NPs_–GO films. The GO Raman spectrum (Figure 2c) exhibits two main broad peaks attributed to the D and G bands appearing at 1350 and 1550 cm^–1^, respectively.^26^ In the case of P3HT_NPs_, the spectrum shows a typical P3HT Raman peak distribution^40^ with a dominant peak located at 1450 cm^–1^, assigned to the stretching vibrational mode of the C_α_=C_β_ bond in the thiophene ring, a smaller peak at 1380 cm^–1^ corresponding to the stretching mode of the C_β_—C_β′_ bond, and a broad peak at 1100 cm^–1^ associated with the C—H vibrational mode^41^ (Figure 2d). In contrast to these rather simple and well-known spectra, the Raman spectrum of the P3HT_NPs_–GO hybrid material presents a wide number of vibrations that emerge as a result of the interactions between the polymer and GO sheets. In the lower-frequency region (<1200 cm^–1^), several small peaks can be observed, such as those located at 724 and 683 cm^–1^, which are related to the C_α_—S—C_α’_ deformation, or the peaks at around 600 cm^–1^, which are attributed to P3HT ring deformations. Additionally, the peak situated in P3HT_NPs_ at 1100 cm^–1^ is shifted to 1140 cm^–1^. Importantly, a new peak arises at 1280 cm^–1^, which can be ascribed to the symmetric C_α_=C_α′_ inter-ring stretching vibration.^42^ This suggests an intensity redistribution of the P3HT C=C and C—C stretching modes, indicative of the existence of pronounced quinoid distortions. Moreover, in the higher-frequency region, the presence of two new sharp and intense peaks can be observed. The ones located at 1521 and 1624 cm^–1^ are usually not visible in P3HT chains and are assigned to the C_β_=C_β′_ stretching and the quinoid structure vibration, respectively.^41^ Moreover, the signals typically associated with P3HT are also present, albeit their intensities and positions are uncommonly modified. In this sense, the intensity of the dominant peak at 1450 cm^–1^, characteristic of P3HT_NPs_, is noticeably less significant. Moreover, the small peak at 1380 cm^–1^ is now shifted to 1400 cm^–1^ and its intensity has notably increased. These results are in good agreement with theoretical studies carried out on sexithiophene oligomers by Mansour et al.^43^ In their work, the calculated data for the vibration associated with the C_α_=C_β_ bond in a radical cation of sexithiophene (6T^**·**+^) also exhibits a shift toward lower energies and a change in the intensities of the related peak. These findings were explained by an increased contribution of the quinoid form to the polymer structure, coinciding with the results in our P3HT_NPs_–GO hybrid material. Please note that in contrast to the hybrid sample, the Raman spectrum of P3HT_NPs_ simply mixed with GO does neither exhibit shifts nor new peaks, indicative of interactions between the two components (see the Supporting Information, Section 4). Therefore, the differences in the Raman spectra of the P3HT_NPs_–GO hybrid and P3HT_NPs_ point toward a major deformation of the P3HT chains. The clear appearance of the peaks associated with the quinoid structure and the activation of vibrational modes, which are generally not observed, except under high-doping and surface-enhanced Raman spectroscopy (SERS) conditions,^41,42^ reveal that the intimate interaction between P3HT_NPs_ and GO is modifying the bond structure of the polymer chains from an aromatic to a quinoid conformation. Furthermore, this kind of conformational change typically has been associated with the existence of polaronic states in P3HT.^43,44^

**Figure 3:**
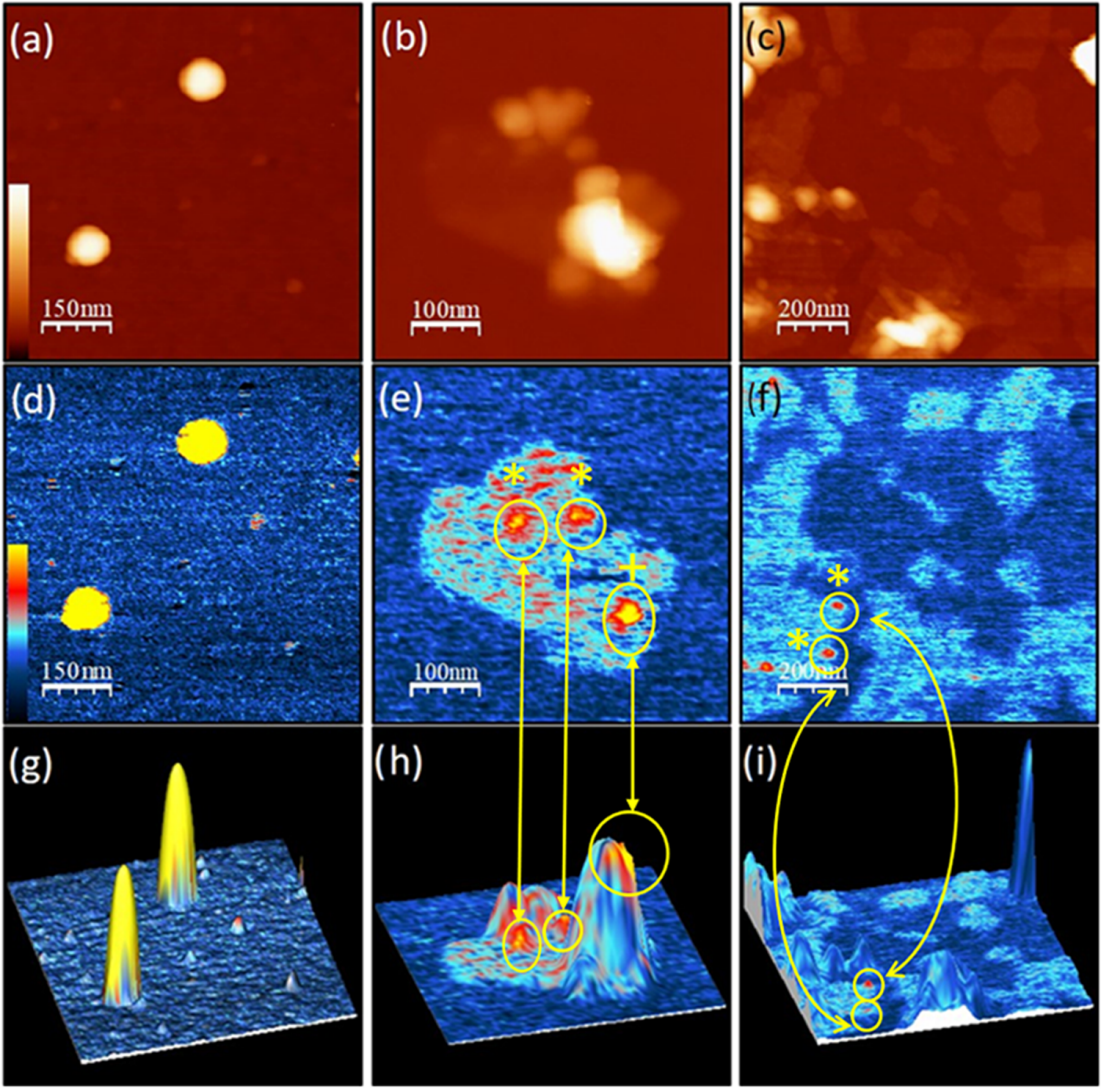
(a)–(c) AFM topography images: *z* scale = 50 nm. (d)–(f) KPFM images (*z* range = −50 (black) to 200 mV (yellow) with respect to the silicon substrate) of P3HT_NPs_ (left column)_,_ P3HT_NPs_–GO nanohybrids (middle column), and P3HT_NPs_–GO nanohybrids together with pristine GO (right column) deposited on a silicon substrate. (g)–(i) Combined topographic and SP data, merged into a single 3D image such that the topographic data is represented as a 3D relief, while the SP data is superimposed as a color map. Circles marked with * and ^+^ in (e, f, h, i): zones representing high SP value and low topography (no correlation) and a zone of high SP value and high topography exhibiting a slight displacement (no direct correlation), respectively.

More insight into the electronic effects caused by the structural deformations taking place in the hybrid material is obtained by combined atomic force microscopy (AFM) and Kelvin probe force microscopy (KPFM) studies probing the morphology and the surface potential (SP) of P3HT_NPs_–GO nanohybrids, respectively. Samples were carefully prepared from ultradiluted dispersions drop-cast onto p-type silicon substrates (see the Experimental Section), thus ensuring to probe the nanohybrids as isolated objects. Figure 3 shows the AFM images of single, isolated P3HT_NPs_ (left column), the P3HT_NPs_–GO hybrid material (middle column), and the P3HT_NPs_–GO hybrid material codeposited with pure GO flakes (right column). First, the morphology of the samples according to the topography images is discussed. The sample of individual P3HT_NPs_ (Figure 3a) is composed of round nanoparticles exhibiting a well-defined shape with diameters and heights of about 50–100 and 20–40 nm, respectively. The negligible tip convolution for these samples suggests that the nanoparticles are flattened, most likely due to strong interactions of P3HT with the silicon substrate. The morphology of the P3HT_NPs_–GO nanohybrids (Figure 3b,c) is in good agreement with the TEM observations, clearly showing that the P3HT_NPs_ are located on the surface of GO flakes. The lateral size of the P3HT_NPs_ on the GO flakes is about 50 nm, typically smaller than that of individual P3HT_NPs_, while showing a much broader lateral size and height distribution. On smaller GO flakes, usually a single P3HT_NPs_ is found (see the Supporting Information), whereas on larger GO flakes, the density of P3HT_NPs_ is quite high, eventually forming aggregates. Importantly, no isolated P3HT_NPs_ are observed on the sample, implying that the P3HT nanoparticles are attached quite strongly to GO as a consequence of the applied miniemulsion process.

The KPFM images (Figure 3d–f) show the surface potential (SP) of the corresponding samples, more precisely, the local difference of the Fermi Level with respect to the tip used for imaging, whereby more positive KPFM values (brighter image colors) correspond to more positively charged regions, as explained in detail in the supporting information of a previous publication.^31^ To simplify data interpretation and the discussion, in the following, all SP values are given with respect to the silicon substrate, serving as an internal standard in this work. The KPFM images of isolated P3HT_NPs_ (Figure 3d) show an excellent correlation of topography and surface potential, exhibiting an SP difference of +350 ± 50 mV for P3HT_NPs_. By contrast, the KPFM measurements of the P3HT_NPs_–GO nanohybrids (Figure 3e,f) reveal a notably distinct response. First, only a few of the P3HT_NPs_–GO nanohybrids show a clear SP difference as compared to the mean SP value of GO flakes (ΔSP_GO_ = +50 ± 20 mV, light blue color), while most of the nanoparticles exhibit essentially the same SP as GO. Second, for those few nanoparticles that display a clear SP difference with respect to GO, their highest values do not surpass +150 mV (for detailed profiles, see Figure S5 in the Supporting Information). The different behavior is seen particularly well in the combined 3D representation of topography and surface potential (Figure 3g–i), which allows a precise colocalization of topographic and surface potential data. Importantly, these images show that surface potential values are not directly correlated with surface morphology. In particular, high surface potential (red-yellow color) is not always associated with the highest topographic regions of the P3HT_NPs_ on the GO flake, as clearly indicated by the circles in the image. Even more intriguing, some regions showing P3HT_NPs_ located on GO display essentially the same SP as GO. This is especially evident in the sample composed of pure GO flakes codeposited with the P3HT_NPs_–GO nanohybrids (Figure 3i). On this sample, the same SP is measured on (a) pristine GO (which can be considered a local “reference potential for GO”), (b) on the part of the isolated nanohybrid not covered by P3HT_NPs_, and (c) most parts of the isolated nanohybrid covered by P3HT_NPs_. It thus appears that most of the P3HT_NPs_ located on the GO flakes in the hybrid sample are transparent in the KPFM images. Since this facilitates only the observation of the surface potential of the underneath GO sheet, it suggests a lack of free charge carriers in the P3HT_NPs_ of the hybrid material. This rather complex electronic behavior of the nanohybrids implies that the P3HT_NPs_ are not simply adsorbed onto the GO sheets but experience a strong interaction involving the modification of their electronic structure. Most likely, this is related to the enhanced stabilization of the quinoid chain conformation of the P3HTN_NPs_ on GO, as implied by the Raman results, being established upon the in situ miniemulsion process.

**Figure 4:**
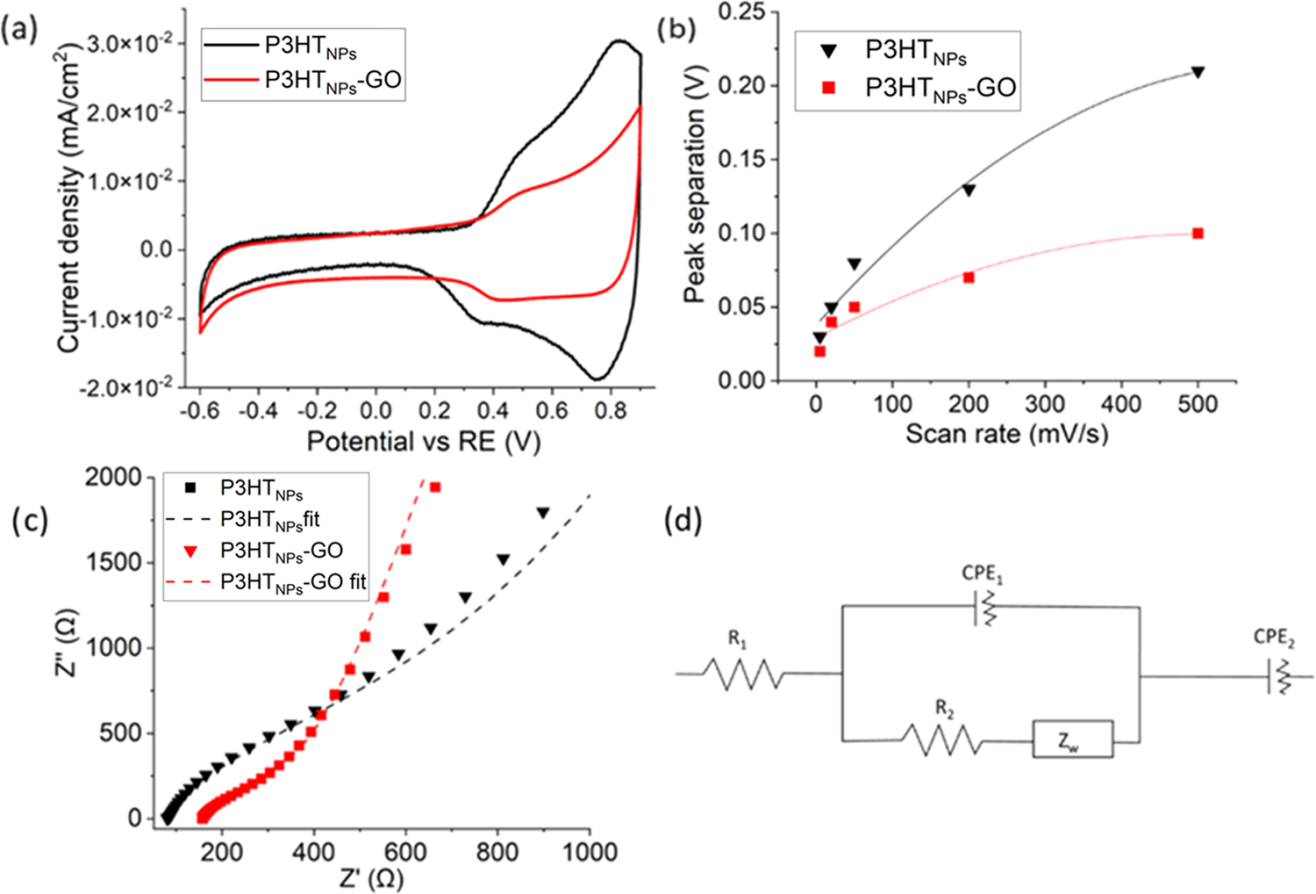
(a) Cyclic voltammograms of P3HT_NPs_ and P3HT_NPs_–GO hybrid films at a scan rate of 200 mV/s. (b) Separation of the redox peaks as a function of the scan rate for P3HT_NPs_ and P3HT_NPs_–GO hybrid films. (c) Nyquist plot of the impedances at 0.45 V. (d) Equivalent circuit employed to fit the impedance data.

To determine the implications of the distinct electronic behavior, electrochemical studies were carried out on P3HT_NPs_ and P3HT_NPs_–GO films. Cyclic voltammetry (CV) experiments performed on P3HT and P3HT–GO films are shown in Figure 4a. The voltammogram displays two oxidation peaks centered at 0.45 and 0.8 V, ascribed to aggregated and amorphous zones of the P3HT, respectively.^45^ The peak centered at 0.45 V is of special interest since p-doping processes of P3HT take place during this oxidation wave and are usually described as the key processes that enable the formation of the polaronic states of P3HT.^13^ As observed in the cyclic voltammograms, the behavior of this oxidation peak at 0.45 V is different in the case of P3HT_NPs_ and the P3HT–GO hybrid. Cyclic voltammetry measurements at different scan rates were performed on P3HT_NPs_ and P3HT_NPs_–GO films (see SI Figure S6a,b) to assess the evolution of the associated redox peaks and thus the kinetic reversibility of the charge transfer processes. It is noticed that the shift of the oxidation and reduction peaks in P3HT_NPs_ films are significantly larger than in P3HT–GO films for the same scan rates (Figure 4b). While the former behavior points to irreversible processes and rather slow electron transfer rates in P3HT_NPs_ films, the scan-rate-independent potential difference for the P3HT_NPs_–GO hybrid films indicates a more Nernstian behavior, enabling a fast electron transfer between the film and the electrolyte solution.^46−48^ The existence of faster charge transfer processes in the P3HT_NPs_–GO hybrid film can be related to the modulation of the P3HT structure caused by the interaction with the GO sheets, in full agreement with the Raman and KPFM observations.

These phenomena were further explored by electronic impedance spectroscopy (EIS) studies, which were carried out at 0.45 V, the oxidation point of P3HT. The noticeable changes in the Nyquist plot (Figure 4c) of both types of materials underline the existence of different electronic processes in the films. The model employed to fit the experimental data (Figure 4d) was developed following a previous work of our group with similar hybrid compounds.^49^ The equivalent circuit consists of resistance *R*_1_ in series with a parallel combination of constant-phase element CPE_1_ (an element used to describe a nonideal capacitor) and resistance *R*_2_ in series with a Warburg impedance, which refers to a finite-length diffusion effect in the film (*Z*_w_).^49^ Finally, a second CPE (CPE_2_) is placed in series with the previous elements. The parameters employed in the equivalent circuit are related to different elements of the electronic system. Thus, resistance *R*_1_ is associated with a resistance inherent to the measurement system (which includes both the electrolyte and electrode). CPE_1_ is associated with the capacitance of the film–electrolyte interface, while *R*_2_ is assigned to the resistance of the charge transfer processes between the film and the electrolyte. The Warburg impedance is attributed to the diffusion of the charged species in the inner part of the film and along the interface between the film and solution, being necessary to equilibrate the charge generated at the electrode. CPE_2_ is an element that refers to the intrinsic electrochemical capacitance of the film. The fitting results (Table 1) reveal that both *R*_2_ and *Z*_W_ are three times lower in the P3HT_NPs_–GO hybrid films compared to the ones in P3HT_NPs_. This clearly underlines the formation of favorable charge transfer processes between P3HT_NPs_ and GO sheets in the hybrid films. This is also in agreement with the decrease of the CPE_2_ value, suggesting that less charge is stored in the P3HT_NPs_ of the hybrid films, as consistently observed in the cyclic voltammogram in Figure 4a, and in full agreement with the KPFM results.

**Table 1:** Fitted EIS Parameters (*R*, *Y*_0_(CPEs and Z_W_), α_1_(CPEs)) According to the Equivalent Circuit in Figure 4d, Whereby *Y*_0_ Expresses the Admittance Value and α is the Capacitance Degree of the CPEs

	*R*_1_ (Ω)	*R*_2_ (Ω)	*Y*_0_(CPE_1_) (S)	α_1_(CPE_1_)	*Y*_0_(Z_w_) (S)	*Y*_0_(CPE_2_) (S)	α_2_(CPE_2_)
P3HT_NPs_	82.1	782	3.4 × 10^–5^	0.88	3.2 × 10^–4^	7.9 × 10^–5^	1
P3HT_NPs_-GO	156.3	210.4	3.7 × 10^–5^	0.84	1.2 × 10^–3^	4.2 × 10^–5^	0.94

As stated above, the oxidation processes occurring at positive potentials are typically associated with the appearance of the polaronic states in the polymer. When P3HT is exposed to potentials higher than 0.45 V (vs Ag/AgCl), it undergoes a p-doping process, which entails the formation of positively charged polymer chains and the change in the main conformation in the bond structure along the P3HT to a more quinoid-like form (Figure 2d).^50^ All of these conformational and electronic alterations usually induce polaronic states, noticed as bands in the NIR appearing on the cost of those in the UV–vis range.^50^ This effect forms the base for electrochromic applications in P3HT-based materials.^50^ We thus carried out electrochromic studies to further analyze the charge transport behavior in our P3HT_NPs_–GO hybrid material.

**Figure 5:**
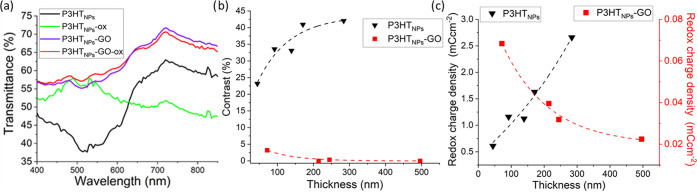
Electrochromic studies. (a) UV–vis transmittance spectra of P3HT_NPs_ and P3HT_NPs_–GO films with similar thicknesses in neutral and oxidized states. (b) Variation of the contrast as a function of the film thickness. (c) Redox charge density of the films as a function of the film thickness.

The transmittance spectra of the two types of films in their neutral and oxidized states (Figure 5a) reveal a clear change of the peak at 510 nm, indicating the transition from the neutral to the oxidized state (between 0 and 0.6 V) in P3HT_NPs_. The former (neutral state) tends to absorb in the visible range of the spectra, while the latter (oxidized state) presents its transitions in the NIR.^51^ In contrast, in the P3HT–GO hybrid, the transmittance spectrum remains almost constant even after the oxidation process.

The electrochromic performance of the films of both materials is evaluated at λ_max_ = 510 nm (the wavelength corresponding to the maximum transmittance modulation), providing the contrast (transmittance variation between neutral and oxidized states) as a function of the film thickness (Figure 5b). Two distinct tendencies can be observed for the P3HT_NPs_ and the P3HT–GO hybrid. In the case of P3HT_NP_, the change in transmittance at 510 nm between neutral and oxidized states increases with the thickness of the film until a maximum is reached (in this case, for a film of ∼300 nm), as typically observed in conductive polymeric systems.^51−53^ However, in the case of the P3HT_NPs_–GO hybrid, the electrochromic response is almost negligible, even for the thicker films. This unexpected observation is in sharp contrast to previous studies on the influence of GO on the electrochromic effects of P3HT.^54^ A directly related observation refers to the thickness-dependent charge density of the films. While P3HT_NPs_ films show an enhancement of charge density with increased film thickness, typical for conductive polymers, the P3HT_NPs_–GO nanohybrid exhibits the opposite behavior, offering advantages when prepared as very thin films (Figure 5c). Additionally, the observation that redox charge densities for P3HT_NPs_–GO films are two orders of magnitude lower compared to those obtained in P3HT_NPs_ films confirms a notable loss in redox activity, as previously mentioned. Therefore, the unexpected loss of electrochromic effects in the P3HT_NPs_–GO hybrid films combined with the unusual thickness dependence indicates the suppression of the characteristic polaronic charge transport process in the nanohybrid films, associated with the established P3HT_NPs_–GO interface interactions favored by the more planar quinoid conformation of the polymer chains in the P3HT_NPs_ upon their formation in the miniemulsion process.

## Conclusions

In this study, we report on the formation of a P3HT_NPs_–GO nanohybrid through a miniemulsion process employing GO as a surfactant. We demonstrate that the presence of GO in this process favors the formation of a quinoid conformation of the P3HT polymer chains, enabling an intimate contact of the P3HT_NPs_ to the underlying GO sheets. Our studies in the liquid phase, on individual objects and on supported films, consistently reveal that the established interface interaction between P3HT_NP_ and GO facilitates an effective channel to extract charges from the P3HT_NPs_ in the hybrid material, thereby suppressing the polaronic charge transport processes in the polymer chains of the P3HT_NP_ in the nanohybrid. This unprecedented finding underlines the importance of the applied synthesis approach for controlling interface interactions and establishing favorable charge transport/extraction properties in hybrids of conjugated polymer nanoparticles and sheets of graphene oxide. It further constitutes an important fundamental contribution toward an enhanced understanding of nonpolaronic transport phenomena, a topic of very recent interest in the field of conducting polymers.^55,56^ Moreover, the possibility of achieving effective and rapid charge extraction from P3HT_NPs_ let us envisage the sustainable design of novel high-performance optoelectronic device structures based on water-dispersible conjugated polymer nanoparticles.

## Experimental Section

### Materials

Regio-regular poly(3-hexylthiophene) with 95% RR and MW > 5000, CHCl_3_, and THF were purchased from Sigma-Aldrich.

### Synthesis

For the synthesis of the P3HT_NPs_–GO hybrid, P3HT was dissolved in CHCl_3_ (1 mg/mL) and stirred for 20 min. Then, 1 mL of the solution was transferred to 10 mL of water solution containing 1 mg of the desired GO dispersion. The solution was tip-sonicated for 2 min at maximum power (400 W, 24 kHz as the maximum frequency) until it turned into a brown dispersion. Then, the emulsion was heated to 65 °C under vigorous stirring until chloroform was removed and the solution transformed into a transparent purple dispersion. Finally, the as-prepared dispersions were filtrated to remove nondispersed polymer excess. Dispersions obtained with different GO flake sizes were studied (see the S.I., Figure S2), and the most stables were found to be those made with the smallest GO flake dispersion (4 h) consistently employed in this work. P3HT_NPs_ nanoparticles as the control sample was prepared by the reprecipitation process. P3HT was dissolved 1 mg/mL in tetrahydrofuran (THF) and stirred overnight. Thereafter, the solution was mildly heated at 35 °C until the color changed to bright orange. Then, 1 mL of P3HT solution was rapidly injected into 10 mL of deionized water. Obtained dispersions were mildly heated until THF was removed from the solution and the solution remained stable.

### Characterization

For TEM/EDS characterization, original dispersions of the P3HT materials were diluted to a 1:100 proportion and then drop-cast onto lacey carbon TEM grids. TEM and EDS characterizations were performed on a probe-corrected Thermo Fisher Titan-Low-Base 60-300 operating at 300 kV (equipped with a Cs-probe corrector (CESCOR from CEOS GmbH)). EDS data were recorded using an Oxford Instruments Ultim Max TLE 100 spectrometer.

UV–Vis absorption spectra of dispersions were recorded on a Shimadzu UV-2401 PC spectrophotometer. The concentration of the water dispersions was adjusted to acquire an absorbance value of 0.3 at 510 nm. Photoluminescence (PL) emission spectra were recorded on a Horiba Jobin Yvon Fluoromax-P using an absorption wavelength of 510 nm, employing dispersions used in UV–vis spectroscopy using a 10 mm pathlength quartz cuvette.

Samples for PL were measured from the same dispersions with an absorbance value of 0.3 at 510 nm in UV–vis. For Raman analysis, dispersions in water of P3HT_NP_, P3HT–GO, and GO were deposited onto glass substrates by spray coating and measured by Raman spectroscopy using an excitation wavelength of 532 nm by a Horiba Jobin Yvon HRLAB HR 800 UV apparatus.

AFM/KPFM samples were prepared by drop casting of ultradiluted solutions (1:100 with respect to the original solution) of both P3HT_NPs_ and P3HT_NPs_–GO on highly doped p-type silicon (1–10 Ω·cm, Siltronix). Prior to deposition, the surface was cleaned with ethanol to remove organic contaminants and then rinsed with double distilled water; afterward, the surface was exposed to an ozone atmosphere to make the substrate hydrophilic. Directly after deposition, the samples were again rinsed with double distilled water and then mildly heated (between 40 and 50 °C) for about 30 min to evaporate physisorbed water. The measurements were performed with Pt-coated tips (OMCL-AC240TM-R3, *k* = 2 N/m) mounted on a Nanotec SFM system provided with a PLL/dynamic board under ambient conditions (RH = 45%, *T* = 25 °C). The topography images were acquired in the amplitude modulation noncontact dynamic mode (AM-DSFM) with an oscillation amplitude of 10 nm and a tip–sample distance between 7 and 10 nm. The KPFM images were acquired in the frequency modulation mode (FM-KPFM) with *V*_AC_ = 500 mV and a frequency ν_AC_ = 7 kHz. The silicon substrate, probed by the Pt tip, revealed a surface potential of +600 mV. To simplify the discussion, the surface potential values of the nano-objects deposited on the silicon substrates were always provided with respect to the silicon substrate, serving as an internal reference standard in this work.

Electrochemical experiments were performed with an AUTOLAB PGSTAT302N potentiostat. Cyclic voltammetry studies of the hybrids are carried out under nitrogen in a three-electrode cell using 0.1 M NaClO_4_ in dry acetonitrile as a supporting electrolyte. The samples were spray-coated onto FTO glasses and used as working electrodes. Carbon and silver/silver chloride (Ag/AgCl, calibrated at 0.19 V vs NHE) were employed as counter and reference electrodes, respectively. EIS experiments were carried out over the same samples in the 0.1–10^6^ Hz frequency range.

To study their electrochromic properties, aqueous dispersion of P3HT_NPs_–GO and P3HT_NPs_ were used to obtain 4–5 films of increasing thicknesses in each case. Film thicknesses were measured with a Dektak 50 profilometer. Films were deposited via spray coating employing an Iwata Eclipse HP-BC airbrush with nitrogen as the carrier gas (∼0.8 bar) onto heated (120 °C) indium tin oxide (ITO)/glass (8–12 Ω/sq., 7 mm × 50 mm × 0.7 mm, Delta Technologies Ltd.).

Spectroelectrochemical measurements were performed in a three-electrode cell with 0.1 M LiClO_4_ (ACN) as an electrolyte (with Pt wire as a counter electrode and an Ag/AgCl wire as a reference electrode, calibrated at 0.19 V vs NHE) using a Varian Cary 50 UV–Vis spectrophotometer and a BioLogic SP-50 potentiostat. Cyclic voltammetry was also performed (−0.4 to 1.1 V vs Ag/AgCl wire, 50 mV/s). Charge density values of the films were obtained from the integration of the CV curves. Stationary spectra in the visible range were obtained at neutral and oxidized states, and transmittance values at 510 nm were extracted from these spectra.

## Supporting Information Available

Additional information on the synthesis of graphene oxide, stability of water dispersions of P3HT_NPs_–GO using different GO sheet sizes, UV–vis and Raman spectra, AFM/KPFM images of P3HT_NPs_ and P3HT–GO nanohybrids, and cyclic voltammetry results of films of P3HT_NPs_ and P3HT_NPs_–GO as a function of the scan rate (PDF)
