# Supplementary material for: Graphene Oxide: Key to Efficient Charge Extraction and Suppression of Polaronic Transport in Hybrids with Poly (3-hexylthiophene) Nanoparticles
Source: Chem Mater. 2023 Apr 20;35(9):3522–31. doi: 10.1021/acs.chemmater.3c00008 (PMC10173772; doi:10.1021/acs.chemmater.3c00008)
Supplement: Supplementary file 1 — cm3c00008_si_001.pdf [file cm3c00008_si_001.pdf]

SUPPORTING INFORMATION

Graphene Oxide: Key to Efficient Charge Extraction and Suppression of Polaronic Charge Transport in Hybrids with Poly (3-hexylthiophene) Nanoparticles

Eduardo Colom,[†] Javier Hernández-Ferrer,[†] Alejandro Galán-González,[†,‡] Alejandro Ansón-Casaos,[†] Mario Navarro-Rodríguez,[§] Elisa Palacios-Lidón,[§] Jaime Colchero,[§] Javier Padilla,[‖] Antonio Urbina,[⊥] Raul Arenal,[#,∇,○] Ana M. Benito,[†] and Wolfgang K. Maser[*,†]

[†]Instituto de Carboquímica (ICB-CSIC), E-50011 Zaragoza, Spain

[‡]Centro de Investigaciones Científicas Avanzadas, Universidade da Coruña (CICA), E-15008 San Vicenzo de Elviña, A Coruña, Spain

[§]Departamento de Física, Edificio CIOyN, Universidad de Murcia, E-30100 Murcia, Spain

[‖]Departamento de Física Aplicada y Tec. Naval, Universidad Politécnica de Cartagena, E-30202 Cartagena, Spain

[⊥]Departamento de Ciencias e Instituto de Materiales Avanzados y Matemáticas (INAMAT^2), Universidad Pública de Navarra (UPNA), E-31006 Pamplona, Spain

[#]Instituto de Nanociencia y Materiales de Aragón (INMA-CSIC-Univ. Zaragoza), E-50009 Zaragoza, Spain

[∇]ARAID Foundation, E-50018 Zaragoza, Spain

[○]Laboratorio de Microscopias Avanzadas (LMA), Universidad de Zaragoza, E-50018 Zaragoza, Spain

*email: wmaser@icb.csic.es

Index

- S1. Synthesis of Graphene Oxide
- S2. Stability of water dispersions of $P3HT_{NPs}$–GO as a function of GO sheet size
- S3. UV-vis spectra
- S4. Raman spectra of $P3HT_{NPs}$ mixed with GO $(P3HT_{NPs}–GO)_{mix}$
- S5. AFM/KPFM images of $P3HT_{NPs}$ and P3HT–GO nanohybrids
- S6. Cyclic Voltammetry of films of $P3HT_{NPs}$ and $P3HT_{NPs}$–GO as a function of scan rate
- S7. References

S1. Synthesis of graphene oxide

Graphite oxide was prepared using a modified Hummers method.[1,2] Specifically, 5 g of graphite flakes were put into a mixture of 170 ml H_2SO_4 and 3.75 g $NaNO_3$, cooled by an ice bath. After stirring for 30 min, 25 g of $KMnO_4$ is slowly added. The reaction was kept at 0 °C for another 30 min. Thereafter, the ice bath was removed, the mixture was warmed up to 35 – 40 °C and stirred overnight. The reaction was terminated by slowly adding 250 ml of deionized water and then 20 ml H_2O_2 (30%) solution. The resulting dispersion was filtered, and the obtained powder material was repeatedly washed with 400 ml of HCl: H_2O (1:10 v/v) to remove any metal ions followed by washing with deionized water until neutral pH was obtained. Finally, the graphite oxide obtained was dried at room temperature. Graphene oxide was obtained by bath sonication (45 kHz) of an aqueous graphite oxide dispersion (2 mg/ml) for 30 minutes, obtaining a brown-colored dispersion. The sheet size of GO was modified via tip sonication fracture of the original GO flakes[3] at 200 W and 15 kHz frequency. GO dispersions of 1 mg/mL were sonicated for three different times (1, 2, and 4 h). Dynamic light scattering (DLS) measurements of the dispersions of the as-prepared and sonication treated GO sheets were performed to establish the hydrodynamic diameter as a measure of the GO sheet sizes (Figure S1).

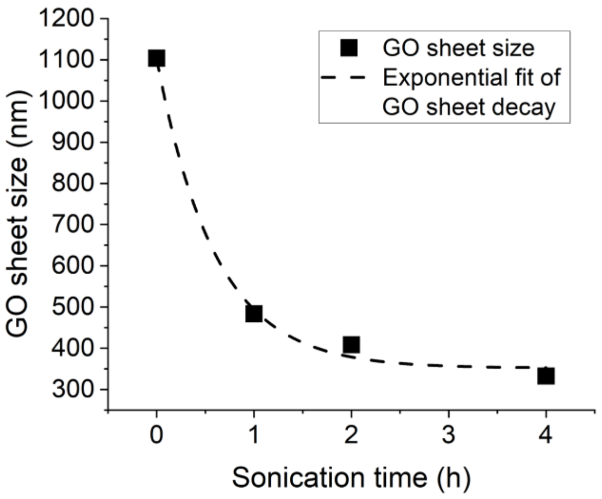

Figure S1. Size of the GO sheets after different sonication times as measured by DLS.

S2. Stability of water dispersions of $P3HT_{NPs}$–GO as a function of GO sheet size

The miniemulsion process was carried out with GO of different sheet sizes (see section above). Importantly, depending on the GO sheet size, the resulting $P3HT_{NPs}$–GO water dispersions show different degree of stability, as indicated by the colors of the resulting dispersions (Figure S2). The most stable $P3HT_{NPs}$–GO hybrids are those leading to dark colored dispersions, obtained by employing the smallest GO sheet sizes (4h sonication) On the contrary, $P3HT_{NPs}$–GO dispersions prepared with the larger sheet sizes on which the $P3HT_{NPs}$ are deposited during the synthesis process reveal a low stability and tend to precipitate and thus the resulting dispersions, due to the loss of $P3HT_{NPs}$–GO material reveal much lighter colors. Therefore, in this work only the stable $P3HT_{NPs}$–GO hybrid materials obtained with the smallest GO size were employed.

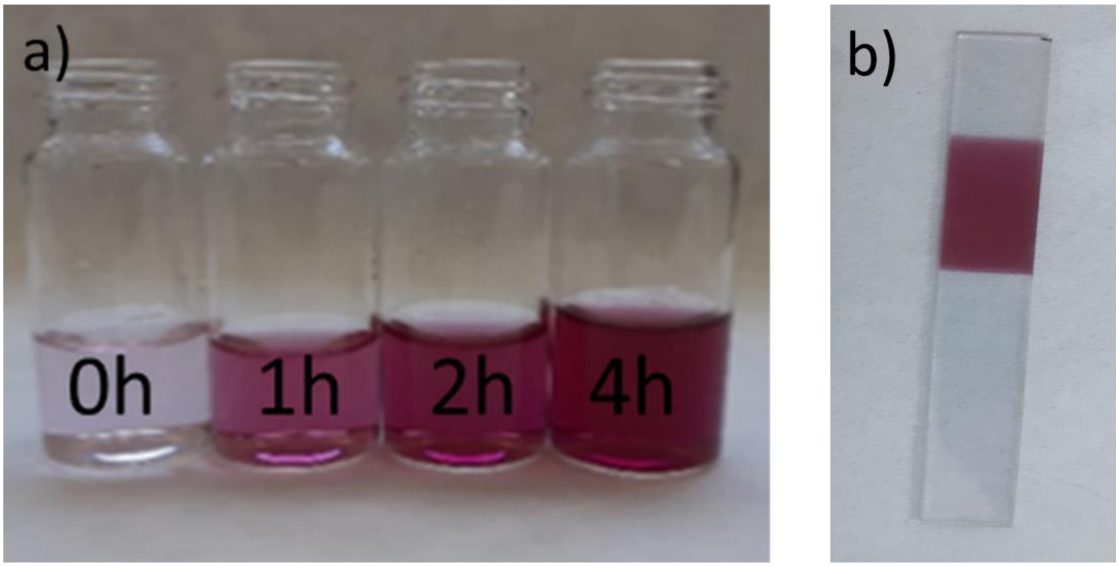

Figure S2. (a) $P3HT_{NPs}$–GO dispersions with different GO sizes (non-sonicated, 1, 2, and 4 h of sonication) from left to right. (b) Homogeneous spray-coated film of $P3HT_{NPs}$–GO hybrid (using 4 h sonicated GO) on a glass substrate.

S3. UV-vis spectra

The original UV-vis spectra of $P3HT_{NPs}$ and $P3HT_{NPs}$–GO are shown in Figure S3.1. The spectra reveal a significant scattering background, characteristic for the presence of P3HT nanoparticles and GO in the respective $P3HT_{NPs}$ and $P3HT_{NPs}$–GO dispersions. For each of the two dispersions, the background scattering between the low-wavelength absorption minimum at about 350 nm and the high-wavelength absorption base at about 700 nm is fitted and subtracted from the respective spectra.

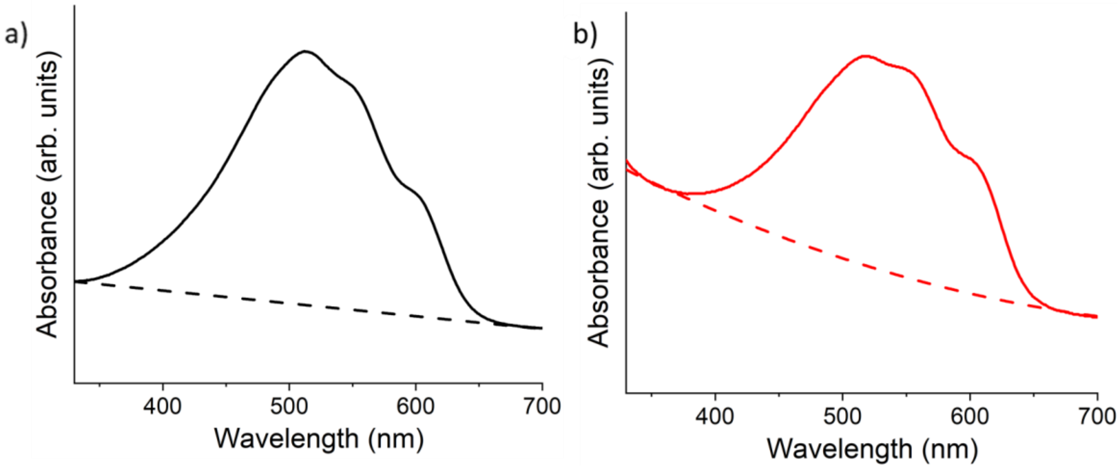

Figure S3.1 UV-vis absorption spectra (straight line) and baseline correction (dotted line) for (a) $P3HT_{NPs}$ and (b) $P3HT_{NPs}$–GO dispersions.

The background corrected spectra, normalized to its maxima, i.e. the intensity of the A_{0-2} fundamental vibronic mode, are shown in Figure 2a of the main manuscript. From these spectra, the values for the intensity ratio of the A_{0-0} to A_{0-1} transitions are determined as 0.55 and 0.65 for the $P3HT_{NPs}$ and $P3HT_{NPs}$–GO samples, respectively. Exhibiting values below 1, this clearly indicates an internal polymer chain structure, typical for the formation of H-aggregates, characterized by interchain coupling interactions. However, the increase of the ratio by almost 20 % for $P3HT_{NPs}$–GO reveals either a decrease in the

interchain stacking and/or an enhanced degree of intrachain coupling, most likely facilitated by a higher degree of planarity of the polymer chain backbone.[4]

From the intensity ratio of the A_{0-0} to A_{0-1} transitions, the free exciton bandwidth W of the aggregates, and the nearest-neighbor interchain Coulombic coupling constant J_0 ($W=4J_0$) can be calculated according to equation S3.2, assuming a Huang-Rhys factor of 1.[5,6]

$$\frac{IA_{0-0}}{IA_{0-1}} = \frac{(1-0.24W/E_p)^2}{(1+0.073W/E_p)^2} \qquad \textbf{Eq. S3.2}$$

whereby E_p corresponds to the main intramolecular vibration, i.e. C = C symmetric stretch mode with a value of 0.18 eV, dominating the coupling to the electronic transition. The resulting values are summarized in Table S3.1

Table S3.1. Exciton bandwidth W and Interchain Coulombic coupling constant J_0 calculated according to Eq. S3.2 with the A_{0-0} / A_{0-1} intensity determined from the normalized background corrected UV-vis spectra shown in Figure 2a in the main manuscript.

	**IA_{0-0} / IA_{0-1}**	**W (meV)**	**J_0 (meV)**
**$P3HT_{NPs}$**	~ 0.55	~ 160	~ 40
**$P3HT_{NPs}$–GO**	~ 0.65	~ 120	~ 30

The reduced value for the coupling constant J_0 for the $P3HT_{NPs}$–GO sample thus indicates either a decrease in the interchain stacking and/or an increase in the intrachain order, most likely facilitated by a higher degree of planarity of the polymer chain backbone.

S4. Raman spectra of $P3HT_{NPs}$ mixed with GO $(P3HT_{NPs}–GO)_{mix}$

For these measurements, 10 mL of water dispersions of $P3HT_{NPs}$ and GO, both at a concentration of 0.1 mg/mL, were mixed in a 1:1 ratio. Spray-coating the $(P3HT_{NPs}–GO)_{mix}$ dispersion onto a glass substrate resulted in a homogeneous film, which was probed by Raman spectroscopy at an excitation wavelength of 532 nm. The resulting spectrum is shown in Figure S4. For the sake of comparison, the spectra of films of GO and $P3HT_{NPs}$ are equally included.

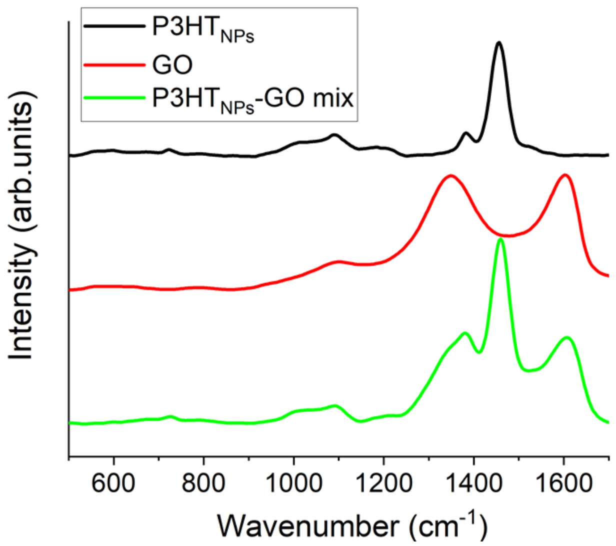

Figure S4. Raman spectra of P3HT, GO and $(P3HT_{NPs}–GO)_{mix.}$ probed at an excitation wavelength of 532 nm.

The $(P3HT_{NPs}–GO)_{mix}$ film sample reveals a spectrum, which is a mere superposition of the characteristic Raman features of GO (D and G band at 1350 cm^{-1} and 1550 cm^{-1}, respectively) and $P3HT_{NPs}$ ($C_α{=}C_ß$ and $C_ß—C_{ß'}$ at 1450 cm^{-1} and 1380 cm^{-1}, respectively). No changes in the peak positions are observable, thus indicating the lack of interactions between $P3HT_{NPs}$ and GO in the $(P3HT_{NPs}–GO)_{mix.}$ sample. This is in strong contrast to the Raman spectra of the $P3HT_{NPs}$-GO hybrid sample (Figure 2c, main manuscript), where significant changes in peak-positions and new peaks are observed,

thus clearly confirming the establishment of strong interactions between $P3HT_{NPs}$ and GO upon the in situ miniemulsion process.

S5. AFM/KPFM images of P3HT$_{NPs}$ and P3HT–GO nanohybrids

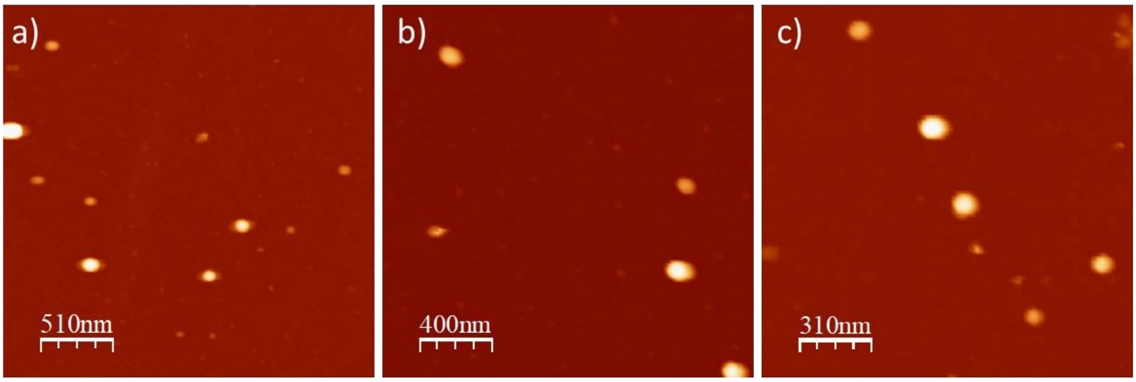

Figure S5.1 AFM topography of different regions with isolated P3HT$_{NPs}$ (z scale=60 nm for all the images).

Statistics from one single image is difficult due to the low concentration of particles, but round particles with lateral size of about 50nm and 100 nm are typically observed presenting a bimodal distribution.

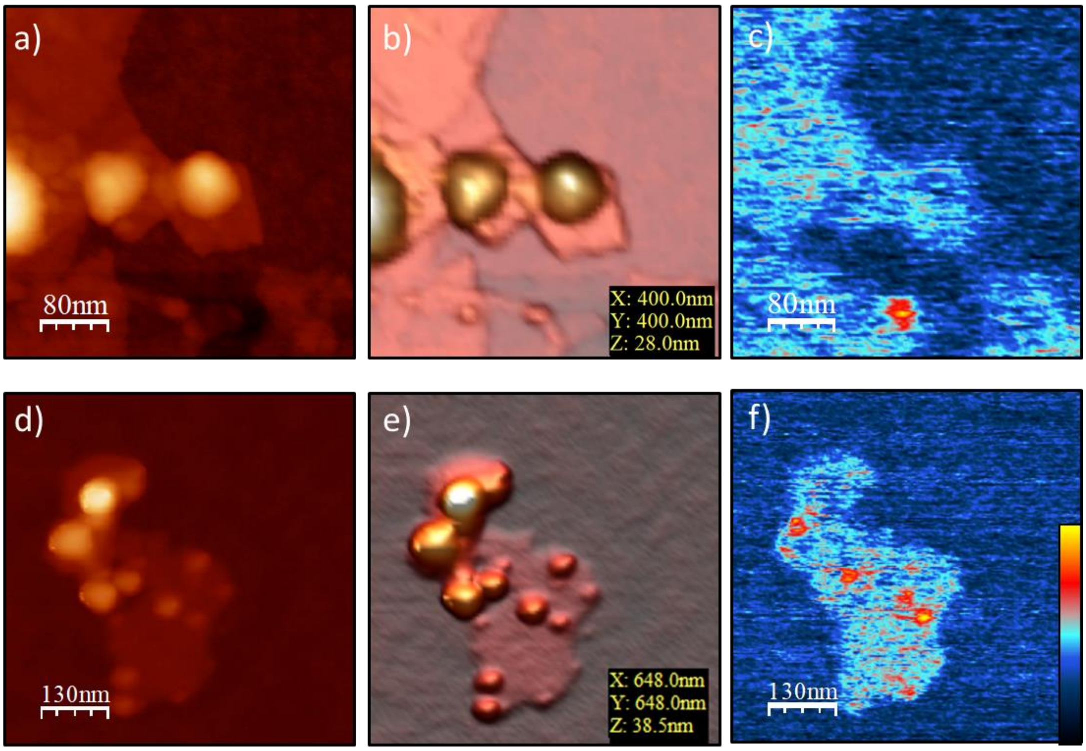

Figure S5.2 a), b) AFM topography of smaller GO flakes with one P3HT particle, c) KPFM image of a). d), e) AFM topography of larger GO flakes with multiple P3HT particles. f) KPFM image of d).

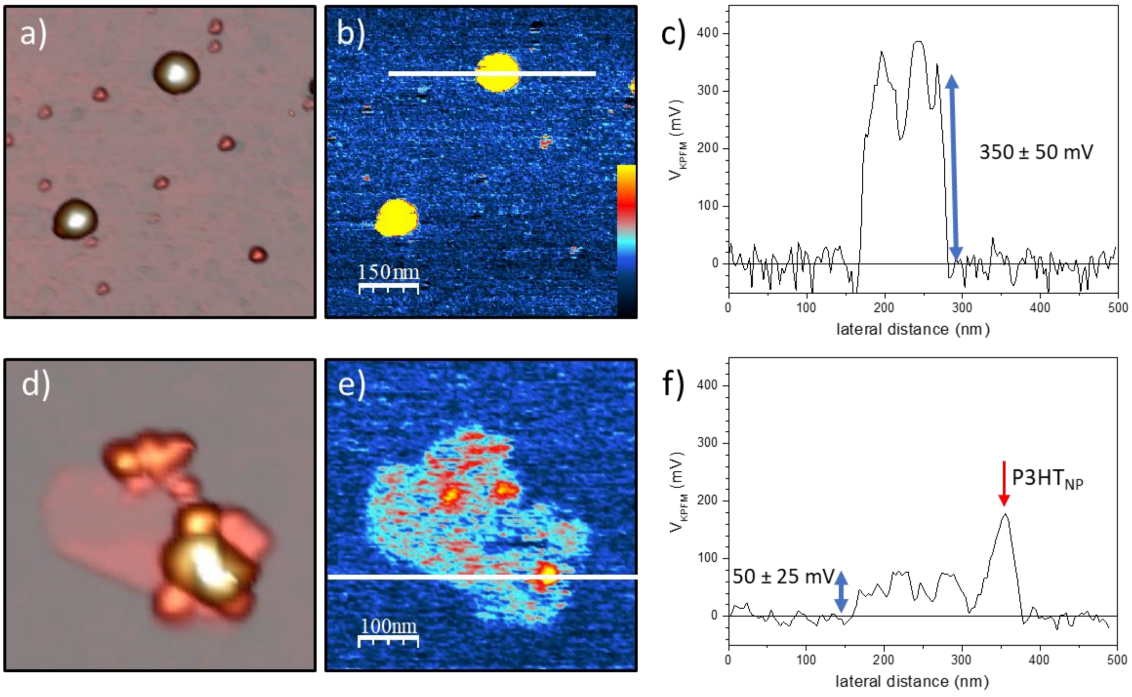

Figure S5.3 AFM topography of a) P3HT nanoparticles and d) $P3HT_{NPs}$–GO nanohybrids. (z scale = 50 nm) b), e) KPFM image (z range = -50 (black) to 200 mV (yellow) with respect to the silicon substrate) of a) and d) respectively. c) and f) line profile of potential of KPFM images b) and e)

S6. Cyclic voltammetry

Cyclic voltammetry of films of $P3HT_{NPs}$ and $P3HT_{NPs}$–GO were performed at different scan rates varying from 5 to 500 mV/s to assess the evolution of the redox peaks related to aggregated zones of P3HT at about 0.45 V of applied potential and thus the kinetic reversibility of the charge transfer processes. Corresponding voltammograms are shown in Figure S3. The resulting potential differences between the oxidation and reduction peaks as a function of scan rate were calculated and represented in Figure 5b of the manuscript.

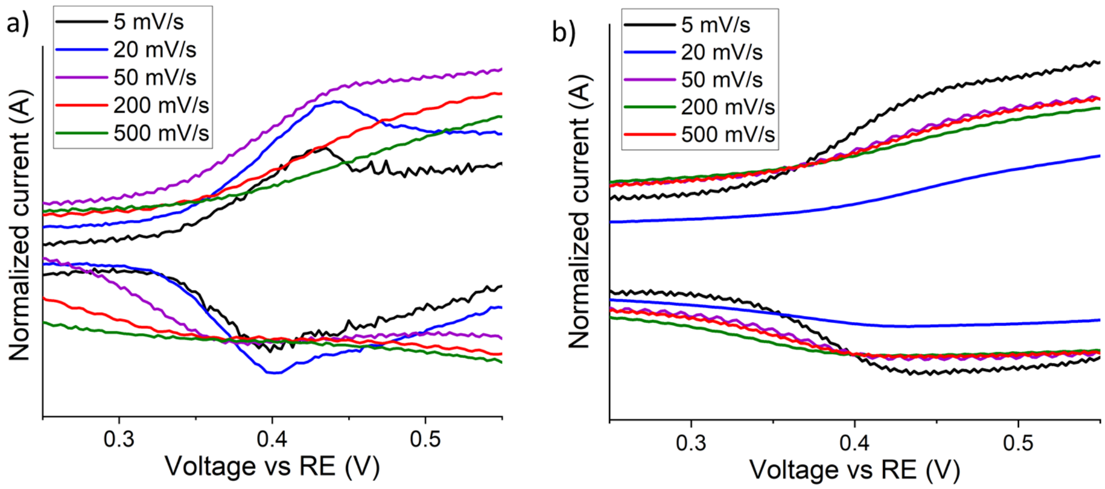

Figure S6. Cyclic voltammetry amplification between 0.25 and 0.55 V for (a) $P3HT_{NPs}$ and (b) $P3HT_{NPs}$–GO at different scan rates

S7. References

(1) Hummers, W. S.; Offeman, R. E. Preparation of Graphitic Oxide. *J. Am. Chem. Soc.* **1958**, *80 (6)*, 1339. DOI: 10.1021/ja01539a017

(2) Vallés, C.; Núñez, J. D; Benito, A. M.; Maser, W. K. Flexible conductive graphene paper obtained by direct and gentle annealing of graphene oxide paper. *Carbon* **2012**, *50 (3)*, 835–844. DOI: 10.1016/j.carbon.2011.09.042

(3) Gonçalves, G.; Vila, M.; Bdikin, I.; De Andrés, A.; Emami, N.; Ferreira, R. A. S.; Carlos, L. D.; Grácio, J.; Marques, P. A. A. P. Breakdown into nanoscale of graphene oxide: Confined hot spot atomic reduction and fragmentation. *Sci. Rep.* **2014**, *4*, 6735. DOI: 10.1038/srep06735.

(4) Nagarjuna, G.; Baghgar, M.; Labastide, J.A.; Algaier, D.D.; Barnes, M.D.; Venkataraman. Tuning Aggregation of Poly-(3-hexylthiohene) within Nanoparticles. *ACS Nano* **2012**, 6 (12), 10750 – 10758. DOI: 10.1021/nn305207b

(5) Clark, J.; Silva, C.; Friend, R.H., Spano, F.C. Role of Intermolecular Coupling in the Photophysics of Disordered Organic Semiconductors: Aggregate Emission in Regioregular Polythiophene. *Phys. Rev. Lett.* **2007**, 98, 206406. DOI: 10.1103/PhysRevLett.98.206406

(6) Clark, J.; Chang, J.-F.; Spano, F.C.; Friend, R.H.; Silva, C. Determining exciton bandwidth and film microstructure in polythiophene films using linear absorption spectroscopy. *Appl. Phys. Lett.* **2009**, 94, 163306. DOI: 10.1063/1.3110904
